# Evaluation of Voice Disorders in Patients with Active Laryngeal Tuberculosis

**DOI:** 10.1371/journal.pone.0126876

**Published:** 2015-05-26

**Authors:** Marcia Mendonça Lucena, Fernanda dos Santos da Silva, Ananda Dutra da Costa, Gabriela Rodrigues Guimarães, Ana Cristina Nunes Ruas, Frederico Pereira Bom Braga, Mateus Pereira Bom Braga, João Gustavo Corrêa Reis, Daniel César Silva da Costa, Mariana Reuter Palmeiro, Valéria Cavalcanti Rolla, Cláudia Maria Valete-Rosalino

**Affiliations:** 1 Laboratory of Clinical Research and Surveillance in Leishmaniasis, Evandro Chagas National Institute of Infectious Diseases, Oswaldo Cruz Foundation, Rio de Janeiro, RJ, Brazil; 2 Departmentof Speech Pathology, Federal University of Rio de Janeiro, Rio de Janeiro, RJ, Brazil; 3 Federal University of Rio de Janeiro, Rio de Janeiro, RJ, Brazil; 4 Laboratory of Clinical Research in Mycobacteriosis, Evandro Chagas National Instituteof Infectious Diseases, Oswaldo Cruz Foundation, Rio de Janeiro, RJ, Brazil; 5 Department of Otorhinolaryngology and Ophthalmology, Federal University of Rio de Janeiro, Rio de Janeiro, RJ, Brazil; Hospital San Agustín. Aviles. Asturias. Spain, SPAIN

## Abstract

**Introduction:**

Laryngeal tuberculosis (LTB) is the most frequent larynx granulomatous disease. In general there is lung involvement, but in an important proportion of cases you can find LTB without pulmonary disease. The lesions observed in LTB, such as ulceration and fibrosis, can interfere in the process of voice production. The involvement of the mucous lining of the vocal folds can change their flexibility and, consequently, change voice quality, and the main symptom is dysphonia present in almost 90% of cases.

**Objective:**

To describe the anatomical characteristics and voice quality in LTB patients.

**Material and Method:**

A descriptive cross-sectional study was conducted with 24 patients.

**Result:**

The most frequently affected sites were vocal folds in 87.5% patients, vestibular folds in 66.7%, epiglottis in 41.7%, arytenoid in 50%, aryepiglottic folds in 33.3%, and interarytenoid region in 33.3% patients. We found 95.8% cases of dysphonia. The voice acoustic analysis showed 58.3% cases of Jitter alterations, 83.3% of Shimmer and 70.8% of GNE.

**Conclusion:**

Voice disorders found in active laryngeal tuberculosis are similar to those reported after clinical healing of the disease, suggesting that sequelae and vocal adjustments may install during the active phase of the disease, negatively impacting the process of vocal quality reestablishment.

## Introduction

Tuberculosis (TB) is a contagious infectious disease of chronic evolution caused by *Mycobacterium tuberculosis (M*. *tuberculosis)*,anatomo-pathologically characterized by the presence of granulomas and central caseous necrosis. The transmission occurs predominantly by air and particularly affects the lungs, but can occur in any organ [1].

In Brazil, the Notifiable Diseases Information System [2] reported 70,047 new cases in 2012. According to the World Health Organization (WHO) Brazil occupies the 17^th^ position in relation to the number of cases. The state of Rio de Janeiro presents the highest incidence rate of the disease in the country. In 2011, Rio de Janeiro presented an incidence rate of 72.3 cases, with the emergence of 11,651 new cases [2,3].

According to WHO 2012 report, of 84,137 TB new cases and relapses reported in Brazil (2011), 10,067 (11.97%) were of the extrapulmonary forms of the disease [2]. HIV infection not only changed the epidemiological trend of the disease, but its clinical presentation, raising the incidence of extrapulmonary forms [1]. The most recurrent forms of extrapulmonary TB are: pleural, peritoneal, pericardial, lymphatic, laryngeal, genitourinary, adrenal, bone, meningeal, intestinal, ophthalmic and cutaneous [4].

TB lesions can be observed in the head and neck in 10% cases [5]. Manifestations in this region are predominant in the larynx. Disorders in external and middle ear, tonsils, cervical lymph nodes, pharynx, oral cavity and salivary glands are less common [6].

Laryngeal tuberculosis (LTB) is the most frequent larynx granulomatous disease. In general there is lung involvement but in an important proportion of cases you can find LTB without pulmonary disease[1,7,8,9]. The lesions observed in LTB, such as ulceration and fibrosis can interfere in the process of voice production [1]. The involvement of the mucous lining of the vocal folds can change their flexibility and, consequently, change voice quality, and the main symptom is dysphonia present in almost 90% of cases [8,10]. The objective of the present paper is to describe the anatomical characteristics and voice quality of patients with active LTB.

## Material and Method

A descriptive cross-sectional study was conducted by an interdisciplinary team of infection disease specialists, otorhinolaryngologists and speech therapists at the Evandro Chagas National Institute of Infectious Diseases (INI)-Fiocruz, from 2010 to 2013, in a cohort of 24 patients with LTB diagnosed by the identification of *M*. *tuberculosis* by at least one of the following methods: sputum analysis through direct examination or culture and/or analysis of tissue samples obtained by biopsy laryngeal through direct examination, culture or histopathology with Wade staining technique. This study was approved by the Ethics in Research Committee—Evandro Chagas National Institute of Infectious Diseases under protocol number 09991613.4.0000.5262 and an informed consent form was signed by all the patients.

During anamnesis the patients were asked about the presence of dysphonia and examined by an otorhinolaryngologist with a 70 degree Karl Storz rigid videolaryngoscope (Tuttlingen, Germany) to assess the presence and location of the mucosal lesions. The study of voice quality was performed simultaneously by three speech therapists through:

1- Analysis of vocal auditory perception, through the GRBASI scale ^(11)^ (G = grade of hoarseness, R = level of roughness, B = breathiness, A = asthenia, S = strain, and I = instability) which are classified from 0 to 3, with 0 = no alteration; 1 = slight alteration; 2 = moderate alteration; and 3 = severe alteration [12].

2—Vocal acoustic analysis with VoxMetria software (CTS Informática, PatoBranco, Brasil), with voice recording of all patients in quiet environment, directly in the computer for better voice capture. We used a Plantronix-model A-20 microphone, with a 10 cm mouth-microphone distance, during the emission of the /e/ sustained vowel at normal condition[13].The parameters used in the present study were: Jitter which indicates the variability of the fundamental frequency perturbation in the short term, with normal pattern up to 0.6%; Shimmer, which indicates the variability of the amplitude of the vocal note in the short term and with normal values above 6.5% and measures of Glottal to Noise Excitation Ratio (GNE), which is an acoustic measure to assess noise in a pulse train that is typically generated by the oscillation of the vocal folds, with normal values below 0.5 (dimensionless).

Dysphonia was defined as the hoarseness symptom reported by the patient regardless the specific vocal assessment. Change in voice quality was defined as a modification in the vocal auditory perception analysis and / or in the vocal acoustic analysis.

The frequencies of the categorical variables were estimated. The values of Shimmer, Jitter and GNE did not present normality in the Shapiro-Wilk test. Therefore, the median and interquartile range were calculated for those variables and the mean ± standard deviation were calculated for the age. The Statistical Package for Social Sciences (SPSS) version 16.0 (IBM Company) was used for data analysis.

## Results

We evaluated 24 LTB patients, with mean age of 50.83 years (SD ± 15.68), with 19 (79.2%) males. Twenty two (91.7%) patients presented concomitant pulmonary TB. Of the two patients with laryngeal tuberculosis with no concomitant pulmonary TB, one had a history of previously treated pulmonary TB and the other was diagnosed with tuberculosis for the first time.

Comorbidities were observed in nine patients and, three of them presented association with more than one disease: 3 cases of hypertension, 2 cases of HIV, 2 cases of bronchitis, 1 case of chronic obstructive pulmonary disease, 1 case of diabetes mellitus, 1 case of skin cancer, 1 case of hepatitis C and 1 case of leprosy. Of the 24 patients, 11 (45.8%) were smokers and 17 (70.8%) consumed alcohol.

Table 1 shows the main data of the 24 patients. We found 23 (95.8%) patients with dysphonia and alteration of the vocal quality. The most frequently affected laryngeal sites were: vocal folds in 87.5%; vestibular folds in 66.7%; epiglottis in 41.7%; arytenoid in 50%; aryepiglottic folds in 33.3%; and interarytenoid region in 33.3% patients. The results of the analysis of vocal auditory perception through the GRBASI scale are shown in Table 2.

**Table 1:** Clinical and vocal characteristics of 24 patients with active laryngeal tuberculosis, Evandro Chagas National Institute of Infectious Diseases, Oswaldo Cruz Foundation, 2014.

N°	Gender*	Age	Smoking	Associated pulmonary tuberculosis	Lesion location	Dysphonia	Voice disorder	Grade of hoarseness
1	M	61	No	Yes	vocal folds and vestibular folds	Yes	Yes	Severe
2	M	56	Yes	Yes	vocal folds and vestibular folds	Yes	Yes	Moderate
3	M	19	No	Yes	vocal folds and vestibular folds	Yes	Yes	Moderate
4	M	69	No	Yes	vocal folds	Yes	Yes	Moderate
5	M	34	Yes	Yes	vocal folds, epiglottis, arytenoid and aryepiglottic folds	Yes	Yes	Moderate
6	F	42	Yes	Yes	vocal folds, epiglottis and arytenoid	Yes	Yes	Severe
7	F	82	No	Yes	vestibular folds and aryepiglottic folds	Yes	Yes	Moderate
8	M	64	No	Yes	vocal folds, vestibular folds and interarytenoid region	Yes	Yes	Severe
9	M	28	No	Yes	vocal folds, vestibular folds, epiglottis, arytenoid and interarytenoid region	Yes	Yes	Moderate
10	M	24	Yes	Yes	vocal folds, vestibular folds, epiglottis, arytenoid, aryepiglottic folds and interarytenoid region	Yes	Yes	Moderate
11	M	40	Yes	Yes	vocal folds, vestibular folds, epiglottis, arytenoid, aryepiglottic folds and interarytenoid region	Yes	Yes	Severe
12	M	54	No	Yes	vocal folds, vestibular folds and arytenoid	Yes	Yes	Moderate
13	M	54	No	No	vocal folds	Yes	Yes	Moderate
14	M	65	No	Yes	vocal folds, vestibular folds, epiglottis, arytenoid and interarytenoid region	Yes	Yes	Moderate
15	M	49	Yes	Yes	vocal folds and vestibular folds	Yes	Yes	Severe
16	M	61	Yes	Yes	vocal folds	Yes	Yes	Moderate
17	M	65	Yes	Yes	Epiglottis and aryepiglottic folds	Yes	Yes	Moderate
18	M	65	Yes	Yes	vocal folds, vestibular folds, epiglottis, arytenoid, aryepiglottic folds and interarytenoid region	Yes	Yes	Severe
19	F	45	No	Yes	vocal folds, vestibular folds, epiglottis and aryepiglottic folds	Yes	Yes	Moderete
20	M	51	Yes	Yes	vocal folds	Yes	Yes	Severe
21	F	48	No	Yes	vocal folds	Yes	Yes	Moderate
22	M	61	Yes	Yes	vocal folds, vestibular folds, arytenoid, and interarytenoid region	Yes	Yes	Severe
23	M	30	No	Yes	vocal folds	Yes	Yes	Moderate
24	F	53	No	No	vestibular folds and epiglottis	No	No	No alteration

*M- male; F- female

**Table 2:** Degree of voice perturbation in the GRBASI* scale in 24 patients with laryngeal tuberculosis, Evandro Chagas National Institute of Infectious Diseases, Oswaldo Cruz Foundation, Rio de Janeiro, 2014.

Grade	G		R		B		A		S		I	
	n	%	n	%	n	%	n	%	n	%	n	%
No	1	4,2	1	4,2	1	4,2	24	100	7	29,2	6	25
Slight	0		11	45,8	7	29,2	0		10	41,7	13	54,2
Moderete	15	62,5	12	50	8	33,3	0		7	29,2	5	20,8
Severe	8	33,3	0		8	33,3	0		0		0	

n- number.

*GRBASI scale.

G—hoarseness, R—roughness B—breathiness; A—asthenia; S—strain

**Fig 1:**
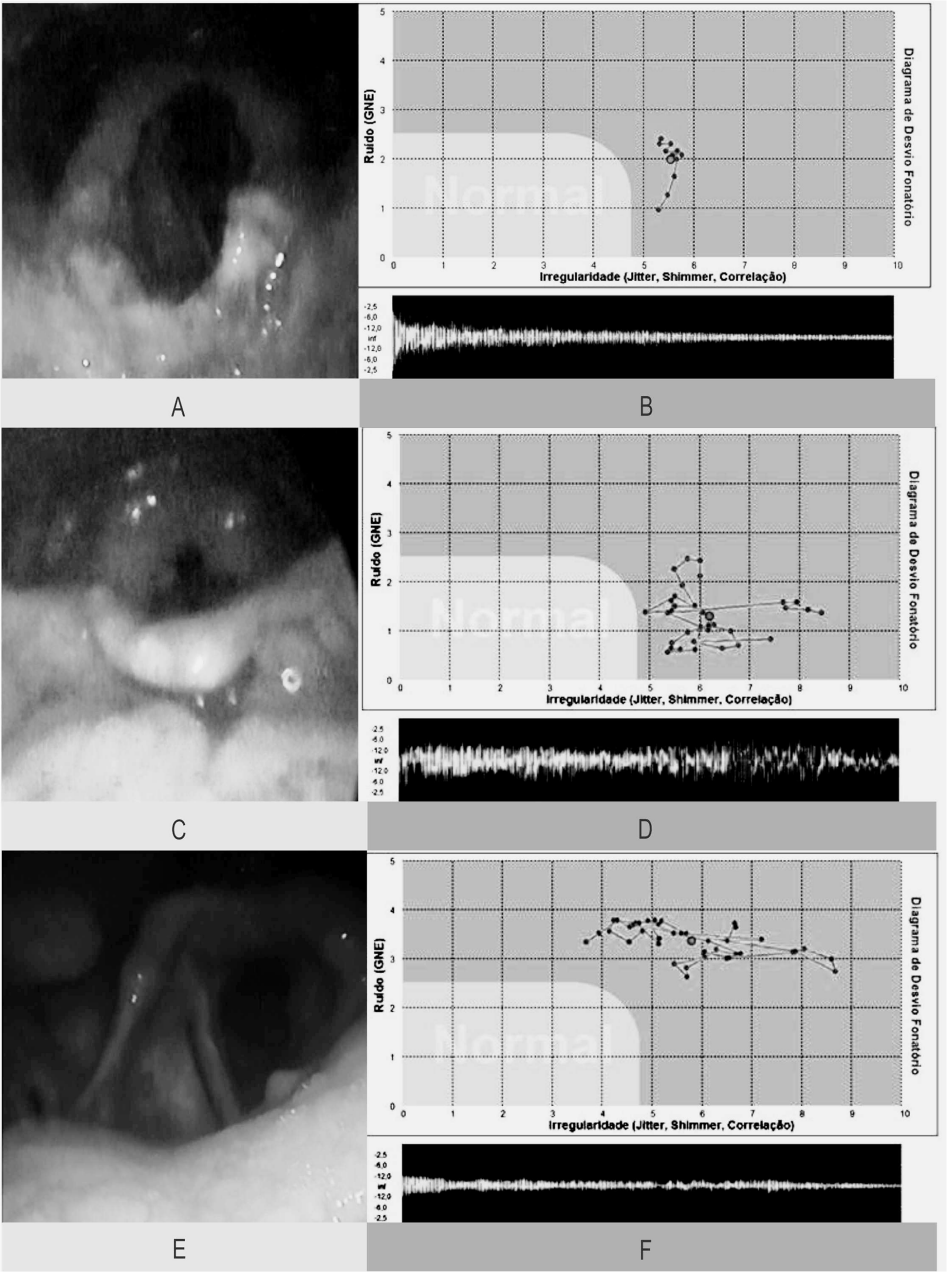
Videolaryngoscopy images and diagrams of phonation deviation in 3 patients with laryngeal tuberculosis, Evandro Chagas, National Institute of Infectious Diseases, Brazil, 2014. *(A) epiglottis with granular appearance with amputation of the edges with uninjured vocal folds (B) phonatory deviation diagram from the normal range (C) granulous infiltrate of the vallecula, necronis area of the epiglottis, infiltration of aryepiglottic folds and arytenoid, precluding visualization of the vocal folds (D) phonatory deviation diagram from the normal range (E) presence of infiltration and hyperemia of the arytenoid, left vocal fold and left vestibular fold (F) phonatory deviation diagram from the normal range.

In the vocal acoustic analysis, 20 (83.3%) patients presented alteration in Shimmer, 14 (58.3%) in Jitter and 17 (70.8%) in GNE. We obtained the following median values: Jitter = 0.97 (IIQ = 0.29–5.55), Shimmer = 10.31 (IIQ = 6.78–18.36) and GNE = 0.31 (IIQ = 0.23–0.51). Fig 1 shows videolaryngoscopy images and the Diagram of Phonation Deviation of three patients.

## Discussion

The anatomical and voice disorders of LTB patients were analyzed. There was prevalence of adult males, as already described in literature [1,7,8,9].The vocal fold was the most involved anatomical site and dysphonia the most frequent symptom, consistent with other studies [7,8,9]. All the patients with lesions in the vocal folds reported dysphonia. The production of a good quality sound depends on the flexibility of the vocal folds and wave formation in the mucous layer and also on a proper and interdependent function of all the muscles acting on its production and the integrity of the vocal tract tissues [11,14]. Therefore, even patients without lesions in the vocal folds may have dysphonia, particularly if they have lesions in the aryepiglottic folds, which are involved in the adduction and abduction processes of the vocal folds. This explains why, of the three patients who did not have lesions of the vocal folds, the only one who had neither dysphonia nor vocal disorder also had no lesions in the aryepiglottic folds.

The grade of hoarseness of patients with voice disorders varied from moderate to severe. The analyses of the alterations found in the GRBASI scale patterns and in the parameters of the acoustic analysis are consistent with the anatomic alterations of the main larynx sites affected, where lesions of the vocal folds and vestibular folds stand out. The alteration of these parameters is directly linked to alterations of the glottal source, such as lack of vibration control and decreased adduction capacity of the vocal folds, reduced glottal resistance and mass lesions in those structures [14].

We did not find in the literature a single report on voice disorder assessment in LTB patients with active lesions. The papers published report functional alterations after medical treatment [13,15]. Considering that voice disorders were still present in more than 80% treated LTB cases [13], we can assume that this alteration begins during the active phase of the disease and is perpetuated due to the LTB lesion scarring process or by functional adjustment mechanisms developed during the phase of voice functional limitation. It is possible that speech-language guidance given to patients during LTB treatment is able to prevent the compensatory mechanisms that lead to the perpetuation of voice disorders, thus restoring the quality of communication and reducing vocal and social impacts of the disease, even before the end of medical treatment.

## Conclusion

Voice disorders found in active laryngeal tuberculosis are similar to that reported in the literature after clinical healing of the disease, suggesting that sequelae and vocal adjustments may install during the active phase of the disease negatively impacting voice quality restoring process.
